# Enhancing livestock vaccination decision-making through rapid diagnostic testing

**DOI:** 10.1016/j.wdp.2019.100144

**Published:** 2019-12

**Authors:** Ashley F. Railey, Felix Lankester, Tiziana Lembo, Richard Reeve, Gabriel Shirima, Thomas L. Marsh

**Affiliations:** a Paul G. Allen School for Global Animal Health, Washington State University, USA; b Boyd Orr Centre for Population and Ecosystem Health, Institute of Biodiversity, Animal Health and Comparative Medicine, College of Medical, Veterinary and Life Sciences, University of Glasgow, Scotland, United Kingdom; c School of Economic Sciences, Washington State University, USA; d Nelson Mandela African Institution of Science and Technology, Tanzania

## Abstract

• Compared to vaccination, the collective approach to diagnostic testing presents a low-fixed cost.
• Existing household livestock-health behaviors increase the likelihood for uptake of preventative health practices.
• Initial evidence to support household investments in livestock preventative health over therapeutic treatments.

## 1 Introduction

Animal vaccination has the potential to indirectly improve household nutrition (Lankester et al., 2015) or translate into investments in human education and health (Marsh, Yoder, Deboch, McElwain, & Palmer, 2016) for the >80 percent of poor livestock-keeping households living in extreme poverty in Africa (Otte et al., 2012). Efforts to encourage animal vaccination target availability issues by training local community health workers to deliver vaccines (Mariner et al., 2012) and promote accessibility through financial incentives that reduce the cost of the vaccine for livestock owners (Jibat, Hogeveen, & Mourits, 2015). Despite these efforts, households underinvest in livestock vaccines. This is due in part to the provision of veterinary goods and services of variable quality and reliability in Africa (Ilukor, 2017), coupled with the unpredictability of adverse health events (Dean et al., 2017), and household responsibilities to invest in competing needs across humans and animals, with direct correlates to human food supply taking precedence (Quinn et al., 2003, Waithanji et al., 2015). Increasing information provision to allow households to more accurately determine whether vaccination is necessary would encourage them to reconsider the benefits to vaccination, including the indirect advancement of household wellbeing.

For decision-making about infectious disease in humans and animals, early disease detection through diagnostic testing offers direct benefits to the individual by providing timely information regarding the necessity for future treatments and costs (Bonner et al., 2015, McKenna and Dohoo, 2006). For a rapid diagnostic test to detect malaria in Africa, the affordability and availability of the test affects demand, but the provision of highly reliable tests may also help reduce the disease burden by signaling to individuals the need for antimalarial treatments (or not) (Cohen, Dupas, & Schaner, 2015). Motivation to apply cost-effective control strategies similarly drive individual households and farms worldwide to use diagnostic testing for infectious disease control in animals (Theurer, White, & Renter, 2015). Testing for the presence or absence of infectious disease in both humans and animals then creates information on treatment needs that may spill over to the entire community. This collective aspect of testing embodies the public good implications of disease control to achieve population immunity while providing others in the community with enhanced decision-making capacities to benefit privately. When disease control strategies that encompass local priorities are necessary to address the emergence of infectious disease (Halliday et al., 2017), assessing whether livestock-dependent households are willing to pay for early disease detection with public good implications offers valuable insight into household-driven control strategies broadly.

Foot-and-mouth disease (FMD) is an endemic, highly contagious viral disease that reduces the productivity of livestock (Knight-Jones & Rushton, 2013). Currently, Tanzanian households mitigate the impacts of FMD with therapeutic antibiotics to treat secondary infections. The limited availability of current and past FMD vaccines in northern Tanzania and the frequent failure to match the vaccine with the circulating diversity of serotypes and sub-types has, historically, induced uncertainty in the protective quality of FMD vaccination (Railey, Lembo, Palmer, Shirima, & Marsh, 2018). Recent research in northern Tanzania, however, demonstrated that, rather than numerous serotypes circulating simultaneously within a region, FMD outbreaks occur in a regular pattern with each sequential outbreak being caused by a different dominant serotype that sweeps slowly across the region (Casey-Bryars et al., 2018). These findings encourage consideration for improved FMD control through a diagnostic technology that enables fast in-situ detection of current serotypes. Early detection would provide time to implement vaccination programs in response to an outbreak with the right vaccine in households sufficiently distant to have not yet been affected. Equipping communities with a field-based mechanism for early detection would enable individual households to make informed disease prevention choices while providing the community with the opportunity to coordinate population-level control.

To examine household preferences for accurate and timely vaccine information delivered through diagnostic testing, we surveyed livestock-dependent households to investigate their willingness to pay (WTP) for a diagnostic test that could indicate which FMD vaccine to apply during an outbreak. We framed the question of willingness to pay such that households contribute to a local fund to purchase the test and have a public veterinarian perform the test on a few, randomly selected infected animals. The collective approach to testing addresses the potential for households and the community jointly to acquire information about the circulating type of FMD to enhance vaccine decision-making for both private and public interests. Households were informed that trained professionals would apply the test, thus, implying an accurate test and widespread, objective dissemination of results to incentivize and assure contributions.

Households provided a stated willingness to pay for diagnostic testing at around 6000 Tsh (USD 2.85). We found low-entry barriers and the provision of the test at a small fixed cost encouraged household willingness to contribute, particularly from households with access to liquid monetary resources and increasing herd sizes. Households exhibiting a strong imperative for livestock health-seeking behavior placed a higher value on the test than those responding to externally organized vaccination campaigns. Finally, we found evidence of a tradeoff between antibiotic usage and preventative health measures, including diagnostic testing to better inform vaccine choices.

We outline these results by first describing the data through a review of the survey setting, sampling procedure, and instrument, followed by presenting the summary statistics and estimation strategy. The paper concludes with the results and discussion on the empirical findings in the context of policy implications for the role of diagnostic testing in reducing the burden of animal disease.

## 2 Data

### 2.1 Setting

**Fig. 1:**
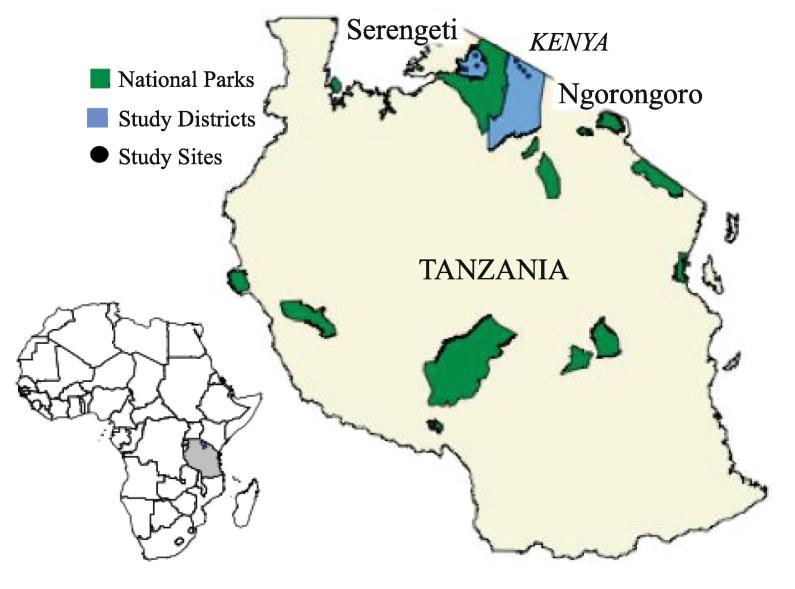
Location of the 10 study sites used for survey data collection across the two study districts.

The study area included two districts of northern Tanzania, Ngorongoro and Serengeti, located between the border of Kenya and the Serengeti National Park (Fig. 1). Agro-pastoralists that engage in agriculture and livestock-keeping income generation activities primarily characterize the Serengeti District. Agro-pastoralists and traditional pastoralists who have been driven to adopt some agro-pastoralist practices (Lankester & Davis, 2016) occupy the study sites in the Ngorongoro District. Similar to agro-pastoralist and pastoralist livelihoods globally, cattle in the study districts remain important to securing nutritional diversity through the production of milk, meat, and other by-products. Cattle also provide economic security in the form of assets, cash from livestock sales, and value-added agricultural production (Randolph et al., 2007). For cattle-owning households, improvements to livestock health impacts household income and wealth, food security, and expenditures on human health and education (Marsh et al., 2016).

FMD not only restricts international trade, but has direct implications for household production, including decreased animal milk yield, high mortality of valuable young stock, lost animal draught power, and closure of or inability to access livestock markets (Perry & Rich, 2007). In Tanzania specifically, households experience significant reductions in milk production and cash from livestock sales, with negative impacts on agriculture potential from losses to traction capacity (Casey-Bryars et al., 2018). Despite the control of FMD through vaccination in some parts of the world (Naranjo & Cosivi, 2013), the presence of four of the seven serotypes with numerous subtypes for each complicates the vaccination process in East Africa. Although the clinical presentation of FMD from each serotype is indistinguishable, protection against these signs depends upon the match of the correct vaccine to the circulating viral serotype (Parida, 2009). Current in-situ diagnostic tests demonstrate presence or absence of FMD (Ferris et al., 2010), but to recognize the serotype causing infection requires laboratory-based diagnostics. However, our hypothetical diagnostic testing scenario attempted to capture preferences for the potential of vaccine matching when the local serotype can be known.

### 2.2 Sampling

We used a multi-stage random sampling design to choose a representative sample of the heterogenous cattle owning population across the two target districts. Based on the estimates of cattle owning populations in the two survey districts (Basic Demographic and Socio-Economic Profile, 2014), we planned to capture six villages and 300 households in the Serengeti District and four villages and 200 households in the Ngorongoro District. Following the selection of logistically feasible administrative wards within the two districts, villages, sub-villages, and households were randomly selected.

To obtain a household sampling frame for each sub-village of households, groups combining village elders, government veterinarians, and village leaders created a list of cattle owning households and then randomly selected households. Enumerators fluent in the local language (Swahili, in addition to either Kuria or Maa) were accompanied by community *balozi* heads or reputable individuals and supervised throughout the survey. Pretesting and data collection for the cross-sectional survey occurred between April 2016 and July 2016. A total of 466 households provided complete responses to the diagnostic testing willingness to pay questions. The remaining households consisted of non-response households and illogical responses. We addressed non-responses by following up with households three times and using cellphones to call absent heads of household. The resulting random distribution of missing values across households, villages, and enumerators lessens the likelihood of systematic bias from non-responders.

### 2.3 Survey instrument

The survey questionnaire contained questions on key variables that may affect household diagnostic testing and vaccination preferences, including household demographics, livestock management practices, and knowledge of and history with FMD. To elicit willingness to pay responses for individual contributions to diagnostic testing, which is a non-market product that has limited history of use in Tanzania, we used a stated-preference approach (Whittington, 1998). Specifically, we used a double-bounded dichotomous choice contingent valuation method (Hanemann, Loomis, & Kanninen, 1991). This is a standard survey approach that can jointly analyze willingness to pay to adopt a product and determine factors underlying adoption behavior. Households were asked if they would contribute to a community fund to have a public veterinarian test for the presence of FMD at the onset of an outbreak so that they knew which vaccine to apply. We set the initial price for each household’s contribution to the fund at 4000 Tanzanian shillings (USD 1.90) after pretesting a number of bid levels and referencing prices for existing livestock health inputs, including vaccines and antibiotic treatments. Households then received the question again with a higher or lower contribution amount depending on the response to the first question. For households that would contribute 4000 Tsh, the contribution amount in the second question randomly varied between 4500 Tsh, 6000 Tsh, and 7500 Tsh (USD 2.15, 2.85, and 3.60). If households said ‘no’ to the initial contribution level, then either 3500 Tsh, 2000 Tsh, or 500 Tsh (USD 1.70, 0.95, or 0.25) was randomly quoted for the lower contribution level. We chose the double-bounded elicitation method over other stated preference approaches (Hansen et al., 2013, Huth et al., 2018) for its cognitive ease on respondents (Alolayan, Evans, & Hammitt, 2017) and gain in statistical efficiency compared to a single question (Hanemann et al., 1991).

We framed the scenario to emphasize that the same information on vaccine quality may be available to the entire community, following the definition of a public good (Tietenberg & Lewis, 2012). The scenario assumes a random selection of only a few cows from the community, instead of testing each and every animal. To elicit truthful responses for a good with public good characteristics, the proposed scenario must be incentive compatible: respondents must care about the outcomes of the event, perceive their actions as consequential, and only value a single event with a likelihood of occurrence. For our scenario, we emphasized the implications of the test to reduce vaccine matching error instead of assuming household familiarity with diagnostic testing technology to enhance the perceived relevancy of the test to the household. We then evoked the perceived consequentiality of household preferences and addressed the likelihood of receiving the test information through an assurance contract (Tabarrok, 1998). Imposing an assurance contract on the provision of the good encourages households to contribute by providing the good if enough households contribute, or assuring the household receives some benefit if others do not contribute. By stating a public veterinarian will apply the test, we assumed delivery by a professional institution with a tradition of providing veterinary services in Tanzania ensures households receive quality and timely vaccine information (Leonard, Bloom, Hanson, O’Farrell, & Spicer, 2013).

The option to privately contribute to the test indicates the presence of strategic responses that may undermine incentive compatibility (Lloyd-Smith & Adamowicz, 2018). Provision bias will occur if households refused to answer the sequence of questions despite valuing the test (scenario rejection) or attempted to increase the likelihood of test provision by responding yes (yea-saying). We accounted for ‘scenario rejection’ by asking an additional question as to why a household would not pay for the test. Fewer than five percent of households presented zero WTP amounts, but none cited reasons unrelated to the value of the test (Mitchell & Carson, 1989). We reduced the chances for ‘yea-saying’ by pretesting a variety of price levels at representative households and consulting local markets for livestock treatment prices (Boyle, Bishop, & Welsh, 1985). For price comparisons, households reported spending around 1000 Tsh (USD 0.50) per cow for substitute livestock health treatments that included antibiotics, with four cows on average treated. The households’ stated preferences for an emergency vaccine applied in reaction to an outbreak and a routine vaccine applied biannually, both offered as private goods per cow, averaged at 5400 Tsh (USD 2.60) and 3900 Tsh (USD 1.90), respectively (Railey et al., 2018). Follow up questions were included as further checks (Section 4a).

### 2.4 Summary statistics

Table 1 presents the descriptive statistics of the data used in the analysis for the full sample and divided between the two districts. We refer to the full sample to describe the average characteristics of the households surveyed, while also providing the descriptions of the variables that are significantly different between the two districts.

**Table 1:** Descriptive statistics for variables relevant to diagnostic testing.

Variable	Reported Averages (St Dev)		
	Full Sample	Serengeti	Ngorongoro
Information Treatment (0 = No; 1 = Yes)	0.09	0.09	0.10
	(0.29)	(0.28)	(0.30)
Income			
*Monthly Off-Farm (≤24,999 Tsh)* (0 = No; 1 = Yes)	0.67	0.56	0.82
	(0.47)	(0.50)	(0.39)
*Monthly Off-Farm (25*–*99,999)* (0 = No; 1 = Yes)	0.15	0.18	0.11
	(0.36)	(0.39)	(0.31)
*Monthly Off-Farm (100,000+)* (0 = No; 1 = Yes)	0.17	0.24	0.07
	(0.38)	(0.43)	(0.26)
*Seasonal Agriculture (≤24,999 Tsh)* (0 = No; 1 = Yes)	0.62	0.58	0.69
	(0.49)	(0.49)	(0.46)
*Seasonal Agriculture (25*–*99,999)* (0 = No; 1 = Yes)	0.12	0.24	0.03
	(0.32)	(0.43)	(0.17)
*Seasonal Agriculture (100*–*499,999)* (0 = No; 1 = Yes)	0.13	0.17	0.08
	(0.34)	(0.37)	(0.27)
*Seasonal Agriculture (500,000+)* (0 = No; 1 = Yes)	0.12	0.07	0.20
	(0.33)	(0.25)	(0.40)
Cattle sold in past year^‡^	5.86	5.12	6.94
	(6.68)	(6.25)	(7.14)
Herd Size^‡^	41.3	36.4	48.5
	(57.5)	(57.2)	(57.3)
Knowledge of diagnostic testing (0 = No; 1 = Yes)	0.31	0.22	0.43
	(0.46)	(0.41)	(0.50)
Low emergency vaccine WTP (0 = No; 1 = Yes)	0.23	0.20	0.26
	(0.42)	(0.40)	(0.44)
Use public vet (0 = No; 1 = Yes)	0.34	0.39	0.29
	(0.48)	(0.49)	(0.45)
Vaccinated in past year (any cattle disease) (0 = No; 1 = Yes)	0.19	0.19	0.23
	(0.40)	(0.40)	(0.42)
Use antibiotics (0 = No; 1 = Yes)	0.79	0.78	0.80
	(0.41)	(0.41)	(0.40)
Vaccinated*Use public vet (0 = No; 1 = Yes)	0.06	0.06	0.06
	(0.23)	(0.23)	(0.24)
Use antibiotics*Use public vet (0 = No; 1 = Yes)	0.30	0.34	0.24
	(0.46)	(0.47)	(0.43)
n	466	275	191

*Notes:* ‡ Reported mean for continuous variables. Values may exceed 100 percent due to rounding.

Households in these districts reported an average of 42 head of cattle per herd, compared to the national average of four cows (Tanzania Livestock Modernization Initiative, 2015). Households in Ngorongoro maintained on average 49 heads of cattle compared to 36 heads of cattle in the Serengeti (p-value <0.05). For both districts, household off-farm monthly income captured income liquidity separate from on-farm production. Around 30 percent of households engaged in off-farm activities, evenly dispersed across earning between 25 and 99,999 Tsh (USD 11.90–47.60) per month and 100,000+ Tsh (USD > 47.60) per month. In the full sample, 45 percent of households received seasonal income from agricultural sales, ranging from 16 percent receiving 25–99,999 Tsh, 13 percent at 100–499,999 Tsh, and 12 percent earning 500,000 Tsh (USD < 11.90, 11.90–238.00, and > 238.00). Around 60 percent of households also reported receiving income from cattle sales with the number of animals sold in the past year ranging from zero to 21 with an average of six animals sold.

Overall, almost 70 percent of households reported FMD outbreaks in the year leading up to the survey, which is consistent with previous accounts of FMD incidence in northern Tanzania (Casey-Bryars et al., 2018). By providing positive responses to a set of knowledge questions, over 30 percent of household’s indicated knowledge of testing for early disease detection ahead of questioning. The Ngorongoro District households demonstrated increased awareness of diagnostic testing compared to those in the Serengeti District (p-value < 0.01). Across both target districts, only 19 percent of households reported vaccinating for any disease in the past year with all of these vaccines provided at a reduced price or for free. However, almost 80 percent of households reported using antibiotics (oxytetracycline, penicillin, or streptomycin) to treat livestock health problems, including secondary infections related to FMD. Use of a public veterinarian for livestock information (35 percent) and low willingness to pay for emergency vaccines (23 percent) were also similar across study districts. To delineate the effects of receiving professional information on uptake of these livestock health practices, we included an interaction between use of a public veterinarian and either antibiotics or vaccination. In the Ngorongoro district, 23 percent of households both used antibiotics and consulted with a public veterinarian compared to 34 percent of households in the Serengeti (p-value < 0.05). For vaccination, only 6 percent of households in both districts also used a public veterinarian. Low vaccination uptake accompanied by the majority of households using antibiotics to treat animal diseases is consistent with existing accounts of animal disease management practices in Tanzania (Caudell et al., 2017).

## 3 Methods and estimation strategy

### 3.1 Conceptual framework

Here we present an illustrative model to motivate our empirical approach, which is not intended to be a complete, definitive model of vaccine decision-making. First consider the vaccination decision. A household either employs a low-input herd maintenance plan (TC_0_) of low-costs and low-productivity, or a household vaccinates for FMD (TC_v_), thereby incurring the additional costs of vaccinating *q* animals with per unit cost C and at a fixed cost *FC*_v_ of acquiring the vaccines.

$${\mathrm{TC}}_{v}=q*C+{\mathrm{FC}}_{v}$$

**Fig. 2:**
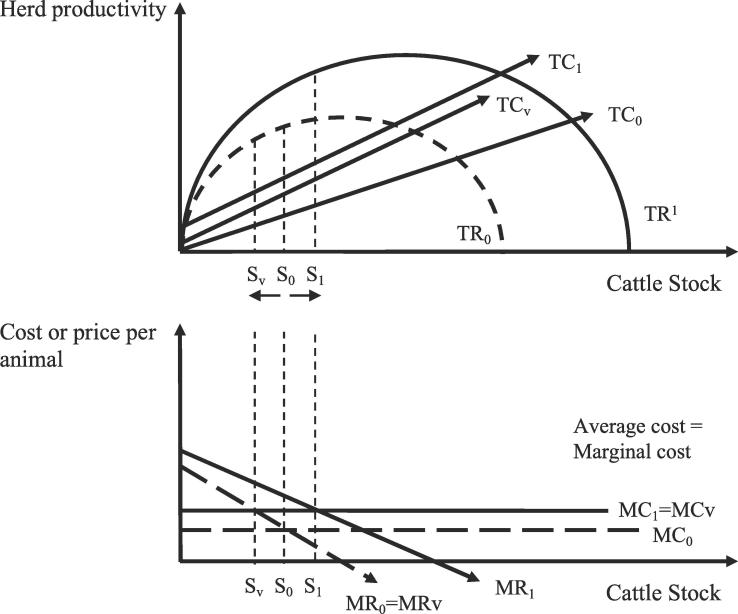
An illustration of the vaccination decision and diagnostic testing decision for this paper. Diagnostic testing adds a fixed cost to the total cost of vaccination (TC_v_ to TC_1_) while increasing total revenue (TR_0_ to TR_1_) for an increase in potential herd profits.

A low likelihood (for example, one in four chance) of receiving a vaccine that protects against the circulating FMD strain suggests households are likely to realize the burden of added vaccine costs without enhancing the productivity of the herd and without realizing additional revenue. Fig. 2 presents this scenario whereby a higher marginal cost (MC_1_) from an animal receiving an ineffective vaccine compared to a low-input plan (MC_0_) reduces the household’s herd profits from S_0_ to S_v_.

In contrast, if the community implements diagnostic test before vaccinating, the protection from a correctly matched and applied vaccine increases the productivity of the herd and total revenue from TR_0_ to TR_1_ while also increasing the total vaccination costs to TC_1_ by a per herd, fixed cost *FC*_d_. Here, diagnostic testing plausibly presents a small fixed cost to the household. The household jointly contributes with the collective group to randomly test a few infected local animals, as opposed to including a variable cost to test each animal in the herd. The total cost is then given by

$${\mathrm{TC}}_{1}={\mathrm{TC}}_{v}+{\mathrm{FC}}_{d}$$

The marginal revenue from securing the correct vaccine matched to the circulating strain before possible disease exposure exceeds the marginal revenue from a low-input plan (MR_1_) and the marginal revenue from an inappropriately matched vaccine (MR_v_) without diagnostic testing (MR_0_). Hence, the shift from S_0_ to S_1_ improves net benefits or profits to the household (Fig. 2).

### 3.2 Empirical approach

Empirically, we followed Hanemann et al. (1991) estimation strategy for a double-bounded sequence of decisions (Appendix, Empirical Estimation). Following expected utility theory, the model assumes a household’s true WTP value falls within the specified intervals from the responses to the first and second contribution levels. We used a maximum likelihood estimator to retrieve the parameter estimates. We then derived the marginal effect on WTP taken at the mean of each parameter and estimated the mean WTP for the overall sample (Hanemann, 1984). The delta method was employed to calculate the confidence intervals around the estimated means (Greene, 2003).

## 4 Results

### 4.1 Robustness checks

Table 2 presents the coefficient estimates from the logistic probability function and the marginal effects from estimating the WTP function. To decide on this model, we first assessed the potential for separate models between the Serengeti and Ngorongoro districts. We tested this by performing individual maximum likelihood estimations for each district separately and a pooled estimation on the districts jointly. The test supports a pooled model instead of individual models for each district, but the Serengeti reports a lower willingness to pay for the test than the Ngorongoro district in the pooled model (-1610 Tsh; USD 0.75).

**Table 2:** Coefficients and marginal effects for variables used in analysis.

Variable	Coefficients	Marginal Effects
Information Treatment (0 = No; 1 = Yes)	0.50	1080
	(0.37)	(7 8 5)
Income		
*Monthly Off-Farm (25*–*99,999)* (0 = No; 1 = Yes)	0.57	1230*
	(0.31)	(6 6 8)
*Monthly Off-Farm (100,000+)* (0 = No; 1 = Yes)	1.02	2190***
	(0.24)	(5 2 6)
*Seasonal Agriculture (25*–*99,999)* (0 = No; 1 = Yes)	0.12	250
	(0.28)	(5 9 3)
*Seasonal Agriculture (100*–*499,999)* (0 = No; 1 = Yes)	−0.54	−1170
	(0.36)	(7 7 0)
*Seasonal Agriculture (500,000+)* (0 = No; 1 = Yes)	0.34	−717
	(0.38)	(8 2 0)
Cattle sold in past year	−0.004	−8.50
	(0.02)	(33.4)
Herd size^†^	0.19	400**
	(0.10)	(2 1 1)
Knowledge of diagnostic testing (0 = No; 1 = Yes)	0.10	221
	(0.22)	(4 7 7)
Low emergency vaccine WTP (0 = No; 1 = Yes)	0.01	25.3
	(0.34)	(7 3 8)
Use government vet (0 = No; 1 = Yes)	−1.83	−3920**
	(0.57)	(1240)
Vaccinated in past year (0 = No; 1 = Yes)	−0.53	−1140*
	(0.29)	(6 1 7)
Use antibiotics (0 = No; 1 = Yes)	−1.36	−2920***
	(0.33)	(7 2 0)
Vaccinated*Use government vet (0 = No; 1 = Yes)	0.89	1920*
	(0.52)	(1130)
Use antibiotics*Use government vet (0 = No; 1 = Yes)	1.52	3250***
	(0.58)	(1250)
Serengeti district (0 = No; 1 = Yes)	−0.75	−1610***
	(0.23)	(4 8 7)
Mean WTP		6110(4 0 6)

*Notes:* n = 466. † log of variable. Standard errors in parenthesis. 2100 Tsh = USD 1

P value: * p < 0.10 ** p < 0.05 *** p < 0.01.

**Fig. A1:**
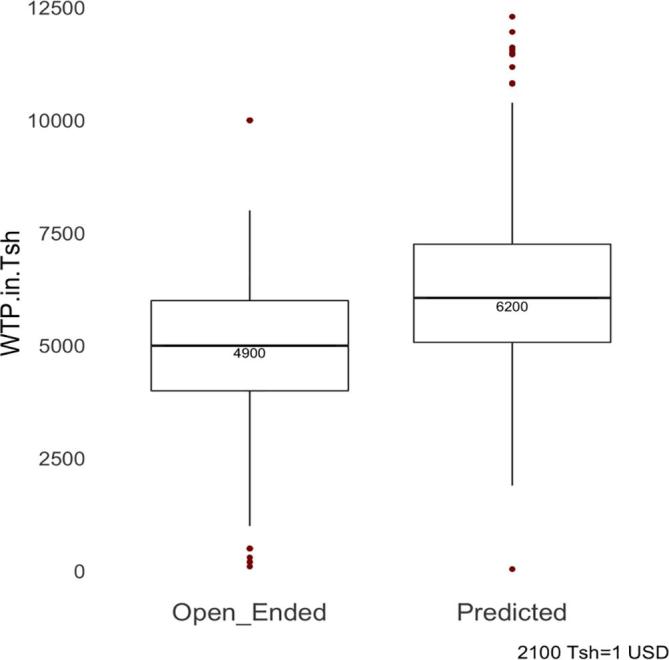
Boxplots of reported household WTP amounts from the open-ended question and the predicted household WTP amounts from the maximum likelihood estimation for diagnostic testing.

The double-bounded stated preference method depends upon households responding to the first and second contribution levels with the same or similar WTP value in mind to ensure consistent responses (Cameron & Quiggin, 1998). We asked households a follow-up, open-ended question on the highest amount the household would pay for the test after the double-bounded sequence of questions to evaluate this. The responses from the follow-up question and the predicted WTP values presented similar distributions, suggesting households answered with the same WTP value in mind throughout the survey (Appendix Fig. A1). Moreover, the probability of a household paying for the test decreased with an increase in price, following theoretical expectations (Mitchell & Carson, 1989) (Appendix Table A1).

The validity of the responses depends on whether households perceived the scenario as consequential. After framing the question to control for strategic responses attributed to scenario rejection based on provision and price (Section 2c), we imposed an information treatment on a portion of the sample (9 percent) to assess potential response bias from unfamiliarity with the circulation of multiple FMD types. All households received a simplified visual of the vaccine matching process, accompanied by a short narrative on FMD, with a randomly selected subset of the households receiving the treatment after the diagnostic testing WTP questions. Resource limitations drove the selection. The selected subset of households served as a preliminary assessment of any potential effects of information on WTP. The insignificant effect of the information treatment suggests households had a basic understanding of the need to match vaccines to circulating types (Table 2).

### 4.2 Marginal effects

Contributions to diagnostic testing reflect household capacity to invest in herd health as a private decision. Total herd size had a positive effect on WTP. For illustrative purposes, as a measure per cow, a household in the first quantile, owning between one and 10 head of cattle, would value the test at 610 Tsh (USD 0.30) per cow. For the top quantile of cattle owning households (>46 cows), the value of the test would equivocate to around 132 Tsh (USD 0.05) per cow reflecting the diminishing marginal value of the test as herd size increases, albeit with a larger total WTP value. Compared to households that received limited off-farm monthly income (<24,999 Tsh), households in the subsequent income levels reported a higher WTP value by 1230 Tsh (USD 0.60) (25–99,999 Tsh) and 2190 Tsh (USD 1.05) (100,000+ Tsh). In contrast, no statistically significant difference in the value of diagnostic testing manifested when comparing households in higher agricultural income levels with those in the lowest level or across the number of cattle sold in the past year.

Existing awareness of diagnostic testing in the absence of current testing failed to predict WTP, but related livestock health practices provided an indication of future adoption preferences. Households that only applied antibiotics reported a lower WTP value than those households not using antibiotics (−2920 Tsh, USD 1.40). Likewise, households that vaccinated in the past year reported a lower WTP for diagnostic testing than those not vaccinating (−1140 Tsh, USD 0.55). Distinguishing low willingness to pay for emergency vaccines from higher willingness to pay values revealed similar valuation of diagnostic testing across the two groups.

Finally, a willingness to consult with health professionals for medical information may also affect individual adoption practices. Acquiring information from a public veterinarian and using antibiotics had a comparatively large marginal effect on WTP for diagnostic testing by 3250 Tsh (USD 1.55) compared to only applying antibiotics or only relying on a public veterinarian or neither. This positive effect extends to vaccination by improving WTP by 1920 Tsh (USD 0.90) for households that vaccinated and benefited from receiving professional advice, as opposed to those who only used one or the other resource or neither. Households that reported receiving information from a public veterinarian without also employing livestock health inputs presented a lower WTP for diagnostic testing than those not using this source (−3920 Tsh, USD 1.90).

## 5 Discussion and conclusions

Our results contribute to growing research on the need for improved information to ensure individually optimal uptake of disease control measures by adding evidence on decision-making at the household level for livestock health inputs that can improve vaccination decisions (Marsh et al., 2016). Compared to vaccination that is per animal, households value diagnostic testing as a fixed cost per herd. The test represents a feasible input for a livestock-dependent household given the cost decreases with each additional animal in the herd. This decreasing marginal value presents a low-entry barrier for households with increasing herd sizes and access to liquid income, as demonstrated by the relationship between WTP and off-farm income. Households need not sacrifice investments in human essentials to afford the test when agricultural decisions and livestock sales may reflect purposeful consumption smoothing choices (Call et al., 2019, Hänke and Barkmann, 2017) but do not strongly influence WTP. That the traditionally pastoralist households in the Ngorongoro district would pay more for the test than the traditionally agro-pastoral communities in the Serengeti further supports testing as an attractive production input for large herds and communities with primarily livestock-keeping households.

A household’s health-seeking behavior influences selection into preventative care interventions. The few households in our study that vaccinated in the past year reported intermittent vaccination with subsidized vaccines offered by either a public or private professional veterinarian (60 percent). These factors suggest existing efforts to signal vaccine quality (Fischer, Karlan, McConnell, & Raffler, 2018), but over limited vaccine availability, which may explain why previous vaccination and preferences for vaccine price failed to depict the complementary relationship between diagnostic testing and vaccination. Simply reporting knowledge about diagnostic testing or reporting use of a public veterinarian also failed to increase household WTP for testing. Instead, households that actively engaged in disease management by concurrently vaccinating or applying antibiotics while also separately seeking livestock health advice from a public veterinarian demonstrated a heightened WTP for testing. Akin to health-seeking behavior for human conditions with a high likelihood of occurrence, these practices may reflect heightened awareness of disease risk (Chinkhumba et al., 2014, Kang et al., 2018) or increased need for quality assured products. Relying on these households to perpetuate diagnostic testing contributions may be problematic if households cluster along behavioral ideologies or physical spaces that cause inequalities in health information access and benefits (Reich, 2018). Ensuring the communal benefits of diagnostic testing additionally requires understanding the distribution of households with differential health behaviors across spatial and temporal patterns of disease incidence.

The benefits of diagnostic testing in animals requires livestock-dependent households to use the test information to make better subsequent decisions that improve household wellbeing (McKenna & Dohoo, 2006). For FMD, diagnostic testing that enhances vaccine matching capabilities may then influence disease incidence, subsequent treatment options, and possible health outcomes through enhanced vaccination decision-making (Hoelzer et al., 2018). To this point, the strong inverse relationship between current antibiotic use and WTP for diagnostic testing suggests a potential substitution effect. When the 70 percent of households that reported FMD primarily treated secondary infections with antibiotics, substitution away from therapeutic antibiotics to diagnostic testing and vaccination would capture a significant market share. Household income may then indirectly benefit from this substitution, as some households reported more than one FMD outbreak per year. Importantly, if interest in early detection signals greater trends in health behavior and veterinary antibiotic use correlates with human health practices, such as sanitation and hygiene (Caudell et al., 2018), reducing the use of veterinary therapeutic antibiotics may benefit the community broadly, including through indirect reductions in transmission of antimicrobial resistance.

To scale up disease control to the community and national level, promoting compliance to early detection and preventative control strategies at the household level is necessary. While households value the prospects of receiving information to enhance vaccine matching capabilities, long-term declines in participation may result (Chandler et al., 2011, Dupas et al., 2016) if the vaccines produce sub-optimal protection despite testing. Similar to human health concerns (Newman-Toker, McDonald, & Meltzer, 2013), illustrating the tradeoffs between vaccination and other treatment strategies and emphasizing when the benefit to vaccinating with testing exceeds the benefit of not testing will provide households with the information to make individually optimal decisions, which may include not vaccinating. Continued interactions with professional health providers further help facilitate this learning process. In Tanzania, the presence of public, private, and community health providers in a decentralized system requires synchronizing the dissemination of information across these sectors and within the communities. This serves to reduce misinformation or delayed information access and overcome household biases to acquire information from one source or another (Closser et al., 2016).

Our study estimates household WTP for the provision of enhanced vaccine information using a hypothetical, community diagnostic test. Using alternative methodologies to assess the value of diagnostic testing characteristics and experiments to evaluate preferences for livestock health provision may elucidate the preferred delivery method of both vaccines and enhanced vaccine information (Howard et al., 2011). Expanding the power of the model through a larger sample size in each district to capture variation within districts or across communities may additionally highlight information inequalities resulting from structural variations and norms (Buntaine et al., 2018, Gottlieb, 2016). Similarly, a complete randomization of the information treatment would extend our ability to infer whether disease control education or information impacts contributions. As is, we only provide initial results suggesting that the information did not significantly impact WTP. Since the survey occurred a number of months after the last FMD outbreak, the immediacy and relevancy of a recent outbreak does not likely bias the results upwards. However, timing data collection periods around outbreaks may improve understandings of the testing process, especially if this can be accompanied by applying diagnostic testing tools in the field with vaccine matching. This then will enhance uptake by providing households with tangible evidence of a complex process.

## 6 Research and ethical clearance

We received permission for field work in the Mara, Manyara, and Arusha regions of Tanzania through the Tanzanian Commission for Science and Technology (COSTECH) permit number 2015 154NA 2015 213, and through the Institutional Review Board at Washington State University, #15091.

## Funding

This work was funded by a prime award (OPP1083453) from the Bill & Melinda Gates Foundation and the Paul G. Allen School for Global Animal Health. The foot-and-mouth disease research platform in northern Tanzania was established through support by the Biotechnology and Biological Sciences Research Council (BBSRC), the Department for International Development and the Scottish Government through the Combating Infectious Diseases of Livestock for International Development initiative (projects BB/H009302/1) and is currently funded by a Merck Animal Health (known as MSD Animal Health outside USA and Canada) grant to the University of Glasgow.

## Author contributions

Conceptualization and design: AFR, FL, TL, RR, TLM; Data acquisition and analysis: AFR, TLM; Writing, reviewing, and editing: AFR, FL, TL, RR, GS, TLM; Supervision: TLM, GS; Final approval: AFR, FL, TL, RR, GS, TLM.

## Declaration of Competing Interest

The authors declare that they have no known competing financial interests or personal relationships that could have appeared to influence the work reported in this paper.

## Figures and Tables

**Table A1:** Probability of responding 'yes' to each bid (in Tsh).

Diagnostic Testing Bid	Probability (%)
500	93
2000	87
3500	76
4000	72
4500	67
5000	62
7500	34

## Appendix

### Empirical estimation

We modeled respondents’ stated preferences for diagnostic testing and vaccine matching information assuming households regard the contributions of others as given and contribute based on perceived individual benefits from testing. We formulate the contribution decision as a function of the test price combination $B_{i}$ presented to household *i*, a vector of observable household characteristics $z_{i}$ and unobservable random characteristics ∊. We assume the parameters enter into a household’s willingness to pay with the relationship :

$$y_{i}\left(B_{i},z_{i}\right)=\alpha +\rho B_{i}+{\lambda \text{'}z}_{i}+{∊}_{i}$$

where α, ρ and λ are unknown parameters to be estimated with a maximum likelihood estimator. We assumed the random error ∊ follows a cumulative logistic distribution with a mean of zero and a variance of $\sigma^{2}$.

We followed Hanemann et al. (1991) estimation strategy for a double-bounded sequence of decisions, assuming a household’s true WTP value $y_{i}$ falls within the specified intervals from the responses to the first $B_{\mathrm{initial}}$ and second contribution levels $B_{\mathrm{low}}$ or $B_{\mathrm{high}}$. A ‘no-no’ response indicated the household WTP lies below the offered contribution price (-∞, B_low_), while a ‘yes-yes’ response suggested the value is either the highest price offered, or above that price [B_high_, ∞). The ‘no-yes’ and ‘yes-no’ responses imply the household’s maximum WTP fell between either of the two amounts [B_low_, B_I_) and [B_I_, B_high_) respectively. A household’s true WTP value can be enclosed within specified intervals to result in four discrete outcomes:

$$Pr\left(y\right)=\left[\begin{array}{c} I_{\mathrm{nn}}ifWTP<B_{\mathrm{low}}\\ I_{\mathrm{ny}}ifB_{\mathrm{low}}\leq WTP<B_{\mathrm{initial}}\\ I_{\mathrm{yn}}ifB_{\mathrm{initial}}\leq WTP<B_{\mathrm{high}}\\ I_{\mathrm{yy}}ifWTP\geq B_{\mathrm{high}} \end{array}\right)$$

whereby $I_{\mathrm{nn}}$, $I_{\mathrm{ny}}$, $I_{\mathrm{yn}}$, $I_{\mathrm{yy}}$are binary-valued indicator variables denoted as one if the argument is true and corresponds to a ‘no-no,’ ‘no-yes,’ ‘yes–no,’ and ‘yes-yes’ response, respectively, or zero otherwise. We model these four outcomes as probability statements using a standard logistic distribution *F()* with mean zero and scale of 1 (Kerr, 2000). The probability statements reflect the difference in indirect utility achieved from either accepting or declining the proposed test contribution level. We then calculated the parameter values that maximize the log-likelihood of observing household *i*’s responses to the first $B_{\mathrm{initial}}$ and second price contribution levels $B_{\mathrm{low}}$ or $B_{\mathrm{high}}$ given the probability statements for each price sequence:

$$L=\sum_{i=1}^{n}\left[I_{\mathrm{nn}}\ln\left(F\left(\alpha +{\rho B}_{\mathrm{low}}+{\lambda \text{'}z}_{i}+{∊}_{i}\right)\right)+I_{\mathrm{ny}}\ln\left(F\left(\alpha +\rho B_{\mathrm{initial}}+{\lambda \text{'}z}_{i}+{∊}_{i}\right)-F\left(\alpha +B_{\mathrm{low}}+{\lambda \text{'}z}_{i}+{∊}_{i}\right)\right)+I_{\mathrm{yn}}\ln\left(F\left(\alpha +\rho B_{\mathrm{high}}+{\lambda \text{'}z}_{i}+{∊}_{i}\right)-F\left(\alpha +B_{\mathrm{inital}}+\lambda \text{'}z_{i}+{∊}_{i}\right)\right)+I_{\mathrm{yy}}\ln\left(1-F\left(\alpha +\rho B_{\mathrm{high}}+\lambda \text{'}z_{i}+{∊}_{i}\right)\right)\right]$$

From this, we derived the marginal effect on WTP taken at the mean of each parameter and estimated the mean WTP for the overall sample (Hanemann, 1984). We used the delta method to calculate the confidence intervals around the estimated means (Greene, 2003). The analysis was done in Stata 13 using the doubleb package (López-Feldman, 2013) and in Gauss 17.

## References

[b0005] Alolayan M.A., Evans J.S., Hammitt J.K. (2017). Valuing mortality risk in Kuwait: Stated-preference with a new consistency test. Environmental and Resource Economics.

[b0010] Basic Demographic and Socio-Economic Profile. (2014). Dar es Salaam, Tanzania. Retrieved from http://tanzania.countrystat.org/fileadmin/user_upload/countrystat_fenix/congo/docs/2012 Tanzania Population and Housing Census-Basic Demographic and Socio-Economic Profile.pdf.

[b0015] Bonner A.B., Monroe K.W., Talley L.I., Klasner A.E., Kimberlin D.W. (2015). Impact of the rapid diagnosis of influenza on physician decision-making and patient management in the pediatric emergency department: results of a randomized, prospective, controlled trial. Pediatrics.

[b0020] Boyle K.J., Bishop R.C., Welsh M.P. (1985). Starting point bias in contingent valuation bidding games. Land Economics.

[b0025] Buntaine M.T., Daniels B., Devlin C. (2018). Can information outreach increase participation in community-driven development? A field experiment near Bwindi National Park, Uganda. World Development.

[b0030] Call M., Gray C., Jagger P. (2019). Smallholder responses to climate anomalies in rural Uganda. World Development.

[b0035] Cameron T.A., Quiggin J. (1998). Estimation using contingent valuation data from a “dichotomous choice with follow-up” questionnaire: Reply. Journal of Environmental Economics and Management.

[b0040] Casey-Bryars M., Reeve R., Bastole U., Knowles N., Auty H., Bachanek-Bankowska K., Lembo T. (2018). Waves of endemic foot-and-mouth disease in eastern Africa suggest feasibility of proactive vaccination approaches. Nature Ecology & Evolution.

[b0045] Caudell M.A., Mair C., Subbiah M., Matthews L., Quinlan R.J., Quinlan M.B., Call D.R. (2018). Identification of risk factors associated with carriage of resistant Escherichia coli in three culturally diverse ethnic groups in Tanzania: A biological and socioeconomic analysis. The Lancet. Planetary Health.

[b0050] Caudell M.A., Quinlan M.B., Subbiah M., Call D.R., Roulette C.J., Roulette J.W., Quinlan R.J. (2017). Antimicrobial use and veterinary Care among agro-pastoralists in Northern Tanzania. PLoS ONE.

[b0055] Chandler C.I.R., Hall-Clifford R., Asaph T., Pascal M., Clarke S., Mbonye A.K. (2011). Introducing malaria rapid diagnostic tests at registered drug shops in Uganda: Limitations of diagnostic testing in the reality of diagnosis. Social Science and Medicine.

[b0060] Chinkhumba J., Godlonton S., Thornton R. (2014). The demand for medical male circumcision. American Economic Journal: Applied Economics.

[b0065] Closser S., Rosenthal A., Maes K., Justice J., Cox K., Omidian P.A., Nyirazinyoye L. (2016). The global context of vaccine refusal: insights from a systematic comparative ethnography of the global polio eradication initiative. Medical Anthropology Quarterly.

[b0070] Cohen J., Dupas P., Schaner S. (2015). Price subsidies, diagnostic tests, and targeting of malaria treatment: Evidence from a randomized controlled trial. American Economic Review.

[b0075] Dean M., Scherr C.L., Clements M., Koruo R., Martinez J., Ross A. (2017). “When information is not enough”: A model for understanding BRCA-positive previvors’ information needs regarding hereditary breast and ovarian cancer risk. Patient Education and Counseling.

[b0080] Dupas P., Hoffmann V., Kremer M., Zwane A.P. (2016). Targeting health subsidies through a nonprice mechanism: A randomized controlled trial in Kenya. Science.

[b0085] Ferris N.P., Nordengrahn A., Hutchings G.H., Paton D.J., Kristersson T., Brocchi E., Merza M. (2010). Development and laboratory validation of a lateral flow device for the detection of serotype SAT 2 foot-and-mouth disease viruses in clinical samples. Journal of Virological Methods.

[b0090] Fischer G., Karlan D., McConnell M., Raffler P. (2018). Short-term subsidies and seller type: A health products experiment in Uganda. Journal of Development Economics.

[b0095] Gottlieb J. (2016). Why might information exacerbate the gender gap in civic participation? Evidence from Mali. World Development.

[b0100] Greene W.H. (2003). Econometric Analysis.

[b0105] Halliday J.E.B., Hampson K., Hanley N., Lembo T., Sharp J.P., Haydon D.T., Cleaveland S. (2017). Driving improvements in emerging disease surveillance through locally relevant capacity strengthening. Science.

[b0110] Hanemann M. (1984). Welfare evaluations in contingent valuation experiments with discrete responses. American Journal of Agricultural Economics.

[b0115] Hanemann M., Loomis J., Kanninen B. (1991). Statistical efficiency of double-bounded dichotomous choice contingent valuation. American Journal of Agricultural Economics.

[b0120] Hänke H., Barkmann J. (2017). Insurance function of livestock: Farmer’s coping capacity with regional droughts in South-Western Madagascar. World Development.

[b0125] Hansen K.S., Pedrazzoli D., Mbonye A., Clarke S., Cundill B., Magnussen P., Yeung S. (2013). Willingness-to-pay for a rapid malaria diagnostic test and artemisinin-based combination therapy from private drug shops in Mukono district, Uganda. Health Policy and Planning.

[b0130] Hoelzer K., Bielke L., Blake D.P., Cox E., Cutting S.M., Devriendt B., Van Immerseel F. (2018). Vaccines as alternatives to antibiotics for food producing animals. Part 1: Challenges and needs. Veterinary Research.

[b0135] Howard K., Salkeld G., Pignone M., Hewett P., Cheung P., Olsen J., Roberts-Thomson I.C. (2011). Preferences for CT colonography and colonoscopy as diagnostic tests for colorectal cancer: A discrete choice experiment. Value in Health.

[b0140] Huth W.L., McEvoy D.M., Morgan O.A. (2018). Controlling an invasive species through consumption: The case of lionfish as an impure public good. Ecological Economics.

[b0145] Ilukor J. (2017). Improving the delivery of veterinary services in Africa: Insights from the empirical application of transaction costs theory in Uganda and Kenya. Revue Scientifique et Technique de l’OIE.

[b0150] Jibat T., Hogeveen H., Mourits M.C.M. (2015). Review on dog rabies vaccination coverage in Africa: A question of dog accessibility or cost recovery?. PLoS Neglected Tropical Diseases.

[b0155] Kang S.K., Jiang M., Duszak R., Heller S.L., Hughes D.R., Moy L. (2018). Use of breast cancer screening and its association with later use of preventive services among medicare beneficiaries. Radiology.

[b0160] Kerr G.N. (2000). Dichotomous choice contingent valuation probability distributions. Australian Journal of Agricultural and Resource Economics.

[b0165] Knight-Jones T.J.D., Rushton J. (2013). The economic impacts of foot and mouth disease – What are they, how big are they and where do they occur?. Preventive Veterinary Medicine.

[b0170] Lankester F., Davis A. (2016). Pastoralism and wildlife: Historical and current perspectives in the East African rangelands of Kenya and Tanzania. Revue Scientifique et Technique de l’OIE.

[b0175] Lankester F., Lugelo A., Kazwala R., Keyyu J., Cleaveland S., Yoder J. (2015). The economic impact of malignant catarrhal fever on pastoralist livelihoods. PLoS ONE.

[b0180] Leonard D.K., Bloom G., Hanson K., O’Farrell J., Spicer N. (2013). Institutional solutions to the asymmetric information problem in health and development services for the poor. World Development.

[b0185] Lloyd-Smith P., Adamowicz W. (2018). Can stated measures of willingness-to-accept be valid? Evidence from laboratory experiments. Journal of Environmental Economics and Management.

[b0190] López-Feldman, A. (2013). DOUBLEB: Stata module to compute Contingent Valuation using Double-Bounded Dichotomous Choice. Boston, MA. Retrieved from https://ideas.repec.org/c/boc/bocode/s457168.html.

[b0195] Mariner J.C., House J.A., Mebus C.A., Sollod A.E., Chibeu D., Jones B.A., van ’t Klooster, G. G. M. (2012). Rinderpest eradication: Appropriate technology and social Innovations. Science.

[b0200] Marsh T.L., Yoder J., Deboch T., McElwain T.F., Palmer G. (2016). Livestock vaccinations translate into increased human capital and school attendance by girls. Science Advances.

[b0205] McKenna S.L.B., Dohoo I.R. (2006). Using and interpreting diagnostic tests. Veterinary Clinics of North America - Food Animal Practice.

[b0210] Mitchell R., Carson R. (1989). Using surveys to value public goods: The contingent valuation method. Resources for the Future.

[b0215] Naranjo J., Cosivi O. (2013). Elimination of foot-and-mouth disease in South America: Lessons and challenges. Philosophical Transactions of the Royal Society of London.

[b0220] Newman-Toker D.E., McDonald K.M., Meltzer D.O. (2013). How much diagnostic safety can we afford, and how should we decide? A health economics perspective. BMJ Quality and Safety.

[b0225] Otte J., Costales A., Dijkman J., Pica-Ciamara U., Robison T., Ahuja V., Roland-Holst D. (2012). Livestock sector for poverty reduction: An economic and policy perspective – Livestock’s many virtues.

[b0230] Parida S. (2009). Vaccination against foot-and-mouth disease virus: Strategies and effectiveness. Expert Review of Vaccines.

[b0235] Perry B.D., Rich K. (2007). Poverty impacts of foot-and-mouth disease and the poverty reduction implications of its control. The Veterinary Record.

[b0240] Quinn C.H., Huby M., Kiwasila H., Lovett J.C. (2003). Local perceptions of risk to livelihood in semi-arid Tanzania. Journal of Environmental Management.

[b0245] Railey A.F., Lembo T., Palmer G.H., Shirima G.M., Marsh T.L. (2018). Spatial and temporal risk as drivers for adoption of foot and mouth disease vaccination. Vaccine.

[b0250] Randolph T.F., Schelling E., Grace D., Nicholson C.F., Leroy J.L., Cole D.C., Ruel M. (2007). Invited Review: Role of livestock in human nutrition and health for poverty reduction in developing countries. Journal of Animal Science.

[b0255] Reich J.A. (2018). “We are fierce, independent thinkers and intelligent”: Social capital and stigma management among mothers who refuse vaccines. Social Science and Medicine.

[b0260] Tabarrok A. (1998). The private provision of public goods via dominant assurance contracts. Public Choice.

[b0265] Tanzania Livestock Modernization Initiative. (2015). Dar es Salaam, Tanzania. Retrieved from http://livestocklivelihoodsandhealth.org/wp-content/uploads/2015/07/Tanzania_Livestock_Modernization_Initiative_July_2015.pdf.

[b0270] Theurer M.E., White B.J., Renter D.G. (2015). Optimizing feedlot diagnostic testing strategies using test characteristics, disease prevalence, and relative costs of misdiagnosis. Veterinary Clinics of North America – Food Animal Practice.

[b0275] Tietenberg T., Lewis L. (2012). Environmental and Natural Resource Economics.

[b0280] Waithanji E., Wanyoike S., Liani M. (2015). ILRI Discussion Paper 29: The role of gender and other socio- economic factors in the adoption of the contagious bovine pleuropneumonia (CBPP) vaccine.

[b0285] Whittington D. (1998). Administering contingent valuation surveys in developing countries. World Development.

